# The effect of increased ambient temperature on thermoregulatory responses in spinal cord injured people

**DOI:** 10.1186/2046-7648-4-S1-A157

**Published:** 2015-09-14

**Authors:** Tamae Yoda, Katy E Griggs, Christof A Leicht, Victoria L Goosey-Tolfrey

**Affiliations:** 1 Department of International Liberal Arts, Dokkyo University, Soka, Japan; 2 Peter Harrison Centre for Disability Sport, Loughborough University, Loughborough, UK

## Introduction

Individuals with a spinal cord injury (SCI) have impaired autonomic thermoregulation, causing a loss of vasomotor control and sweating below the level of lesion due to the disruption of the sympathetic nervous system. Their change in body core temperature can therefore be more pronounced than in able-bodied individuals when they face thermal challenges, such as experienced in the heat, cold or during exercise. Consequently, for individuals with a SCI it is important to be able to control body temperature with behavioural thermoregulation which is influenced by thermal comfort and sensation. The aim of the present study was to evaluate the effect of increased ambient temperature on thermoregulatory responses and perceptual responses of thermal sensation and comfort in individuals with a SCI.

## Methods

Eight participants with a SCI (4 tetraplegia and 4 paraplegia) and 8 able-bodied (AB) participants volunteered for this study. Participants rested in a chair in an environmental chamber for the duration of the experiment. Ambient temperature in the chamber was kept at 27.5 °C for 20 minutes and was then gradually increased to 40 °C during 50 minutes, increasing by 2.5 °C every 10 minutes. Gastrointestinal temperature was measured by a telemetry pill (T_gi_). Skin temperature at ten sites and skin blood flow (skBF) were measured throughout the experiment. Thermal sensation and thermal comfort were reported by participants every 5 minutes using visual analogue scales.

## Results

T_gi _baseline values were similar between SCI and AB (mean (SD) 37.0 (0.5) °C and 37.1 (0.2) °C for SCI and AB, respectively, p > 0.05). At the end of the experiment, there was no change in T_gi _from baseline in either group (37.2 (0.3) °C and 37.1 (0.3) °C for SCI and AB, respectively, p > 0.05). The forehead temperature in both groups was elevated similarly as the ambient temperature increased (p > 0.05). Thigh and calf temperatures were lower in SCI than in AB throughout the experiment (p < 0.05). Mean skin temperature at baseline was lower in SCI than in AB (32.9 (0.4) °C vs 33.7 (0.5) °C, p < 0.05). Mean skin temperature remained lower in SCI than AB as ambient temperature increased (p < 0.05). Change in skBF at the forehead during heat exposure was similar between the groups (p > 0.05), whilst skBF at the thigh was higher in AB (p < 0.05). Thermal sensation and comfort were similar in both groups from baseline to the end of the heat exposure (p > 0.05). Thermal comfort scores decreased to "uncomfortable" in both groups with increasing ambient temperature.

## Discussion

T_gi _in SCI remained unchanged, however the measured skin temperature sites showed greater variation in SCI than in AB. This may be explained by the lack of autonomic thermoregulation below the level of lesion. Importantly, SCI participants could sense the change of ambient temperature equally well as AB participants. Possibly, the remaining sensate area, that depends on the level of lesion, provides sufficient sensory feedback for thermal sensation. It has been reported that the face has a preferential thermosensitivity to temperature sensation [1], therefore intact sensation of the face in SCI may play an important role to induce thermoregulatory behaviour.

## Conclusion

These data suggest that individuals with a SCI are able to sense the change in ambient temperature, which should allow them to control body temperature behaviourally. Further research is needed to explain how the level of the lesion affects these sensations.
